# Fibrosis-Related Gene Profiling in Liver Biopsies of PiZZ α1-Antitrypsin Children with Different Clinical Courses

**DOI:** 10.3390/ijms24032485

**Published:** 2023-01-27

**Authors:** Jan C. Kamp, Naomi N. Kappe, Carlos Fernández Moro, Jan Fuge, Mark P. Kuehnel, Sabine Wrenger, Tobias Welte, Bart van Hoek, Danny D. Jonigk, Padmini P. S. J. Khedoe, Pavel Strnad, Mikael Björnstedt, Jan Stolk, Sabina Janciauskiene, Antal Nemeth

**Affiliations:** 1 Department of Respiratory Medicine, Hannover Medical School, 30625 Hannover, Germany; 2 Biomedical Research in Endstage and Obstructive Lung Disease Hannover (BREATH), German Center of Lung Research (DZL), 30625 Hannover, Germany; 3 Department of Pulmonology, Leiden University Medical Center, Member of European Reference Network Lung, Section Alpha-1-Antitrypsin Deficiency, 2333 ZA Leiden, The Netherlands; 4 Department of Gastroenterology and Hepatology, Leiden University Medical Center, 2333 ZA Leiden, The Netherlands; 5 Department of Laboratory Medicine, Division of Pathology, Karolinska Institutet, 171 77 Stockholm, Sweden; 6 Institute of Pathology, Hannover Medical School, 30625 Hannover, Germany; 7 Institute of Pathology, RWTH University of Aachen, 52074 Aachen, Germany; 8 Medical Clinic III, Gastroenterology, Metabolic Diseases and Intensive Care, University Hospital RWTH Aachen, Health Care Provider of the European Reference Network on Rare Liver Disorders (ERN RARE LIVER), 52074 Aachen, Germany

**Keywords:** PiZZ deficiency, liver, cholestasis, cirrhosis, Z-AAT polymers, mRNA expression, transcriptome profiling, nCounter Fibrosis Panel, lipid metabolism

## Abstract

PiZZ (Glu342Lys) α1-antitrypsin deficiency (AATD) is characterized by intrahepatic AAT polymerization and is a risk factor for liver disease development in children. The majority of PiZZ children are disease free, hence this mutation alone is not sufficient to cause the disease. We investigated Z-AAT polymers and the expression of fibrosis-related genes in liver tissues of PiZZ children with different clinical courses. Liver biopsies obtained during 1979–2010 at the Department of Paediatrics, Karolinska University Hospital, Sweden, were subjected to histological re-evaluation, immunohistochemistry and NanoString-based transcriptome profiling using a panel of 760 fibrosis plus 8 bile acid-related genes. Subjects were divided into three groups based on clinical outcomes: NCH (neonatal cholestasis, favourable outcome, *n* = 5), NCC (neonatal cholestasis, early cirrhosis and liver transplantation, *n* = 4), and NNCH (no neonatal cholestasis, favourable outcome, *n* = 5, six biopsies). Hepatocytes containing Z-AAT polymers were abundant in all groups whereas NCC showed higher expression of genes related to liver fibrosis/cirrhosis and lower expression of genes related to lipid, aldehyde/ketone, and bile acid metabolism. Z-AAT accumulation per se cannot explain the clinical outcomes of PiZZ children; however, changes in the expression of specific genes and pathways involved in lipid, fatty acid, and steroid metabolism appear to reflect the degree of liver injury.

## 1. Introduction

A wild type of serpin family A member 1 (SERPINA1) gene consists of two alleles, named M, which are responsible for the synthesis of quantitatively and qualitatively normal α1-antitrypsin (AAT) protein. The most frequent abnormal alleles (single amino acid mutations) are S (Glu264Val) and Z (Glu342Lys). The combinations of the M, S and Z alleles give rise to the different genotypes whereas the presence of homozygous Z (PiZZ) is one of the most relevant genotypes in the clinic. The subjects carrying PiZZ genotype have severe deficiency (about 90% loss) in circulating AAT protein due to the aberrant folding of the Z-AAT causing intra- and extra-cellular polymerization. The clinical manifestations of PiZZ AAT deficiency (PiZZ AATD) include liver (intracellular retention of aggregated Z-AAT polymers) and lung (low protective levels of functional Z-AAT) diseases, and, less frequently, skin diseases like panniculitis or ANCA+ vasculitis [1]. The worldwide prevalence of PiZZ genotype shows avariability while the highest distribution seems to be present in Northern Europe. Based on model calculations, the estimated number of affected Europeans is about 125,000 individuals [2]. However, the condition is less frequently diagnosed, indicating that there is a high number of undetected cases [3].

Sveger studied 200,000 infants in 1972–74, identified 127 with PiZZ AATD and followed them up to the age of 18 years. Only 14 developed cholestatic jaundice in infancy and 8 had hepatosplenomegaly and mildly abnormal liver function tests [4,5,6]. These data suggested that disease is not caused by the mutation in SERPINA1 gene alone but by a combination with additional factors.

The liver is the major producer of AAT protein (80–90% of human AAT); therefore, hepatocytes can accumulate a large number of Z-AAT aggregates. Several studies have suggested that individual differences in the ability of hepatocytes to transport Z-AAT polymers out of endoplasmic reticulum for degradation by proteasomes, or differences in hepatocyte degradation pathways, may play a critical role in liver disease development [7,8,9]. Among contributory factors, intrauterine infections, autoimmunity, and cryptic hepatitis B or C infection are also proposed [10]. Based on liver biopsy findings, scientists have proposed that portal fibrosis and paucity of bile ducts are the most common features in PiZZ children who progress to liver cirrhosis and/or require liver transplantation [11,12,13]. So far, none of the suggested hypotheses can explain the enormous variation in hepatic phenotype of PiZZ children [14]. Herein, we examine intrahepatic Z-AAT polymers and apply targeted transcriptome profiling to evaluate fibrosis-related gene expression in retrospectively collected liver biopsies of PiZZ children with different clinical outcomes. 

## 2. Results

### 2.1. Patient Cohorts

FFPE liver biopsy samples were obtained from PiZZ AATD children with similar sex and age distribution but different clinical presentations. All cases were divided into three groups: children with neonatal cholestasis who recovered and remained healthy (*n* = 5, NCH), developed cholestasis, liver failure and were liver transplanted (*n* = 4, NCC), and remained healthy (*n* = 5, including one girl with two biopsies at age of 12 and 19 years, NNCH) (Table 1). As expected, all cases showed PAS-D positive inclusions, except in two boys from the NNCH group. One girl did not show PAS-positive inclusions at age of 12 years, but inclusions were present in the repeated biopsy at age of 19 years. She was investigated at 45 years of age and was found to be healthy. Histological scoring of PAS-D accumulation did not correlate with fibrosis scoring. In general, cases from the NCC and NCH groups showed similar fibrosis score and bile duct proliferation, which was higher as compared to the NNCH group. In line, liver enzymes and bilirubin levels were higher in the NCC relative to the NCH or NNCH groups (Supplementary Table S1).

### 2.2. Immunohistochemistry

**Figure 1:**
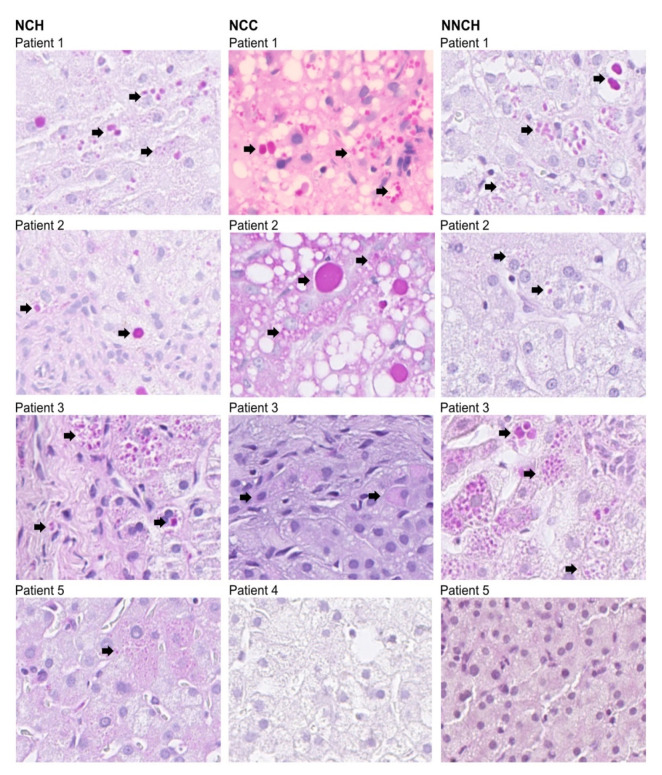
Liver biopsies from PiZZ AATD children (*n* = 12, two samples were not available for imaging, these samples are deposited in archives) subjected to PAS-D staining. Arrows indicate PAS-D positive cells.

**Figure 2:**
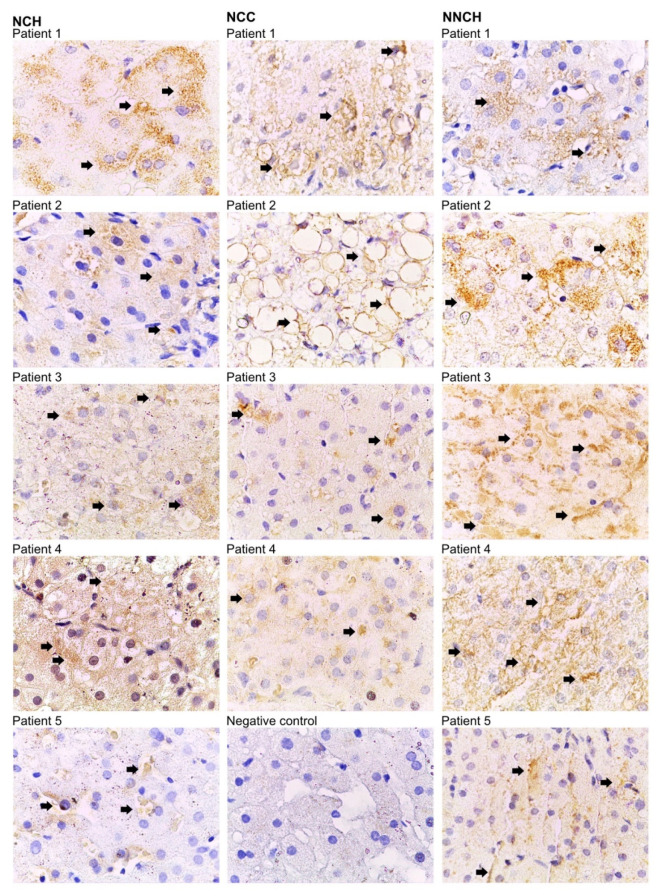
Immunostaining for Z-AAT polymers with D11 antibody. Bound antibodies were visualized using HRP-DAB (3,3′-diaminobenzidine tetrahydrochloride) staining. Arrows indicate Z-AAT polymer-positive areas within hepatocytes (brown color). Images are representative for 7 to 10 taken from different areas of each specimen. Images were taken at 1000-fold magnification using 100x oil immersion objective (Leica). Negative control was performed from patient 1 of the NCH group by omitting the primary anti-Z polymer antibody.

As illustrated in Figure 1 PAS-positive inclusions are typical histological findings in PiZZ AATD [15]. The polymeric AAT was detected by using monoclonal antibody raised against Z-AAT polymers as described previously in [16]. As shown in Table 1, children from the NNCH group did not show more pronounced PAS-D globules and fibrosis, but intracellular Z-AAT polymers were abundant alike in both NCH or NCC groups (Figure 2).

### 2.3. Targeted Liver Gene Profiling

**Figure 3:**
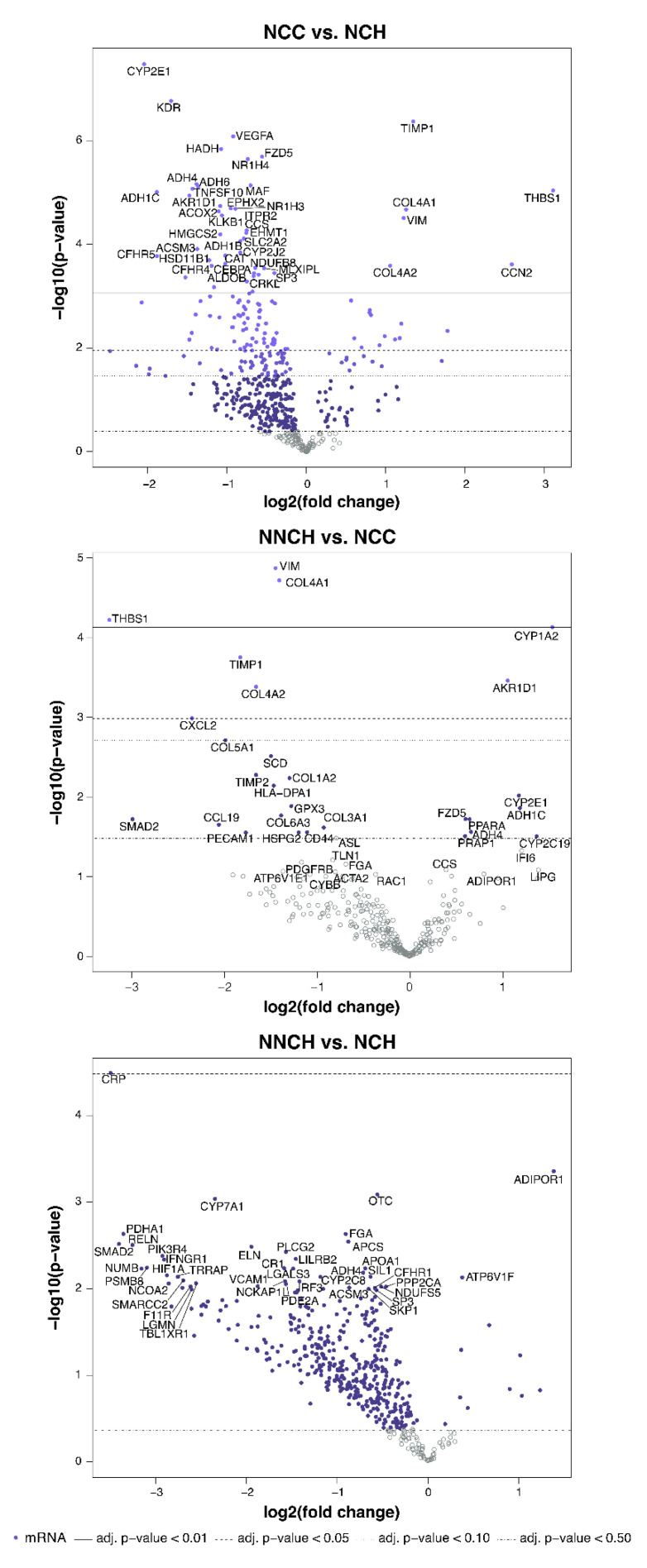
Volcano plots of differentially expressed genes between patient groups.

Transcriptome profiling revealed numbers of differentially expressed genes (DEGs) between PiZZ children’s groups (Figure 3 and Supplementary Table S1). Indeed, the largest number of DEGs was found in NCC versus NCH (50 and 20 genes with decreased and increased expression, respectively) whereas only 7 DEGs were found between NCC and NNCH (5 increased and 2 decreased) and 1 DEG between NCH and NNCH.

**Figure 4:**
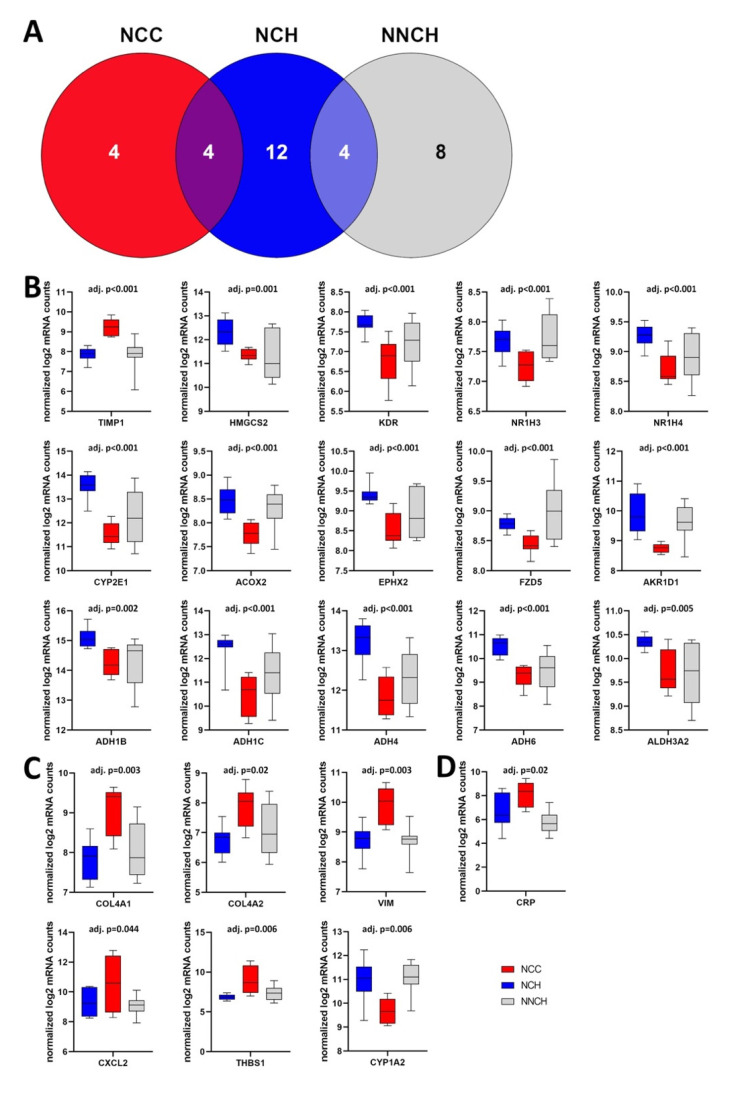
(**A**) Cluster analysis demonstrates a discrimination between the three groups as well as an overlap between those groups with neonatal cholestasis (NCC and NCH) and between those groups with favourable outcome (NCH and NNCH). (**B**–**D**) Box plots of the most significantly different regulated genes in NCC vs. NCH (**B**), NCC vs. NNCH (**C**) and NCH vs. NNCH (**D**). Whiskers show minimum and maximum distribution. *TIMP1*, tissue inhibitor metalloprotease 1; *HMGCS2*, 3-hydroxy-3-methylglutaryl-CoA synthase 2; *KDR*, kinase insert domain receptor; *NR1H3*/4, nuclear receptor subfamily 1 group H member 3/4; *CYP2E1/1A2*, cytochrome P450 2E1/1A2; *ACOX2*, acyl-CoA oxidase 2; *EPHX2*, epoxide hydrolase 2; *FZD5*, frizzled class receptor 5; *AKR1D1*, aldo-keto reductase family 1 member D1; *ADH1B/C*, alcohol dehydrogenase 1B/C; *ADH4*/6, alcohol dehydrogenase 4/6; *ALDH3A2*, aldehyde dehydrogenase 3 family member A2; *COL4A1/2* collagen type IV alpha 1/2 chains; *CXCL2*, C-X-C motif chemokine ligand 2; *THBS1*, thrombospondin 1; *VIM*, vimentin; *CRP*, C-reactive protein.

Given the small sample size, a computer-based hierarchical cluster analysis was performed to confirm a similarity in gene expression within a group and diversity in gene expression between groups. As illustrated in Figure 4A, five clusters were generated whereas one cluster could be generated for 100% of NCH samples, one for 50% of NCC samples, and one for 66% of NNCH samples, respectively. The remaining NCC samples overlapped between NCC and NCH (i.e., those groups with neonatal cholestasis) and the remaining NNCH samples overlapped between NNCH and NCH (i.e., those groups with favorable outcome).

When compared NCC with NCH group, the most significantly higher transcript levels were found for *TIMP1*, *THBS1*, *COL4A1*, and *VIM* whereas *HMGCS2*, *KDR, NR1H3* and *NR1H4*, *CYP2E1*, *ACOX2*, *EPHX2*, *FZD5*, and *AKR1D1*, as well as genes belonging to the alcohol dehydrogenases family like *ADH1B* and *ADH1C*, *ADH4* and *ADH6*, and *ALDH3A2,* were decreased (Figure 4B).

Out of 7 DEGs between NCC and NNCH, an increased expression was found for *COL4A1*, *COL4A2*, *CXCL2* (C-X-C motif chemokine ligand 2), *THBS1*, and *VIM* whereas *AKR1D1* and *CYP1A2* showed a decreased expression (Figure 4C). Interestingly, only one single gene showed an altered expression between NCH and NNCH (i.e., increased expression of *CRP* (C-reactive protein) (Figure 4D).

### 2.4. Activity of Biological Functions

Biological pathway analysis revealed several differentially regulated pathways between groups (Supplementary Table S2). When compared to NCH and NNCH, the NCC group showed decreased activity of many metabolic pathways. These latter include lipid metabolisms (acyl-CoA metabolism, cholesterol metabolism, lipid homeostasis, lipid hydroxylation, lipid metabolism, long-chain fatty acid biosynthetic process, fatty acid metabolic process, omega-hydroxylase P450 pathway, and regulation of lipid transport) and ethanol/alcohol/aldehyde metabolism (alcoholic catabolic process, aldehyde catabolic process, ethanol oxidation). In addition, there was an increased activity of ECM-related pathways (collagen biosynthetic process, ECM organization, and negative regulation of metallopeptidase activity), altered activity of bile acid metabolism and mitochondrial organization, as well as impaired retinoic acid, retinol, and steroid metabolism. In contrast, on the level of biological functions, there were no fundamental differences between NCH and NNCH.

## 3. Discussion

Several studies provided evidence that liver complications in PiZZ AATD arise from the insufficient clearance and accumulation of misfolded Z-AAT polymers within hepatocytes. It is proposed that Z-AAT polymers cause endoplasmic reticular stress and mitochondrial dysfunction. A recent study provided additional evidence that mitochondrial localization of Z-AAT causes mitochondrial dysfunction and liver disease [15]. On the other hand, intrahepatic inclusions of Z-AAT protein are a typical histopathological hallmark of AATD livers [16]. Therefore, one can still question to what extent Z-AAT inclusions (polymers) reflect liver disease evolution and/or liver failure, especially in AATD children.

We hypothesized that higher intrahepatic levels of Z-AAT polymers could segregate children with liver failure from those who remained clinically healthy. In contrast to the direct relationship between Z-AAT polymers and liver damage in adult PiZZ AATD patients [17], we observed no relationship between Z-AAT polymers and clinical outcomes in PiZZ children. Indeed, hepatocytes of NCC and NCH PiZZ children were somewhat less positive for Z-AAT polymers than those of NNCH group. This might be explained in part by the fact that immune reaction(s) and/or autophagy, which clear the hepatocyte from polymers, also causes injury and fibrosis. A similar example can be hepatitis B where not the virus but the immune reaction to the virus causes liver injury [18,19]. Interestingly, results from the transgenic C57BL/6 mouse expressing high levels of human Z-AAT (PiZ mouse) have shown that Z-AAT polymer accumulation is heterogeneous throughout the liver, and that cells with lower Z-AAT protein burdens proliferate better to maintain liver mass. This last may eventually lead to the activation of hepatic stellate cells and hepatic fibrosis [20]. In this scenario, Z-AAT polymer sizes and heterogeneity, as well as the distribution within hepatocytes, may play important dual roles, which remain to be fully elucidated. 

Earlier studies failed to show that the simultaneous occurrence of PiZZ genotype with other genetic liver diseases, like hereditary hemochromatosis or chronic intrahepatic cholestasis, determines the diversity in clinical outcomes in PiZZ children [21]. Defective metabolism of bile acids was proposed as a contributing factor in PiZZ-related liver disease. Bile acid synthesis and excretion comprise the major pathway of cholesterol catabolism and the main feature of enterohepatic circulation. At least two main causes of liver injury can be associated with errors of bile acid metabolism. The first relates to the failure to make “normal” (choli-, chenodeoxicholic and ursodeoxycholic) bile acids, which are a major form of bile secretion. The latter will lead to impaired bile flow and reduced hepatic excretion of the biliary components, such as cholesterol and other lipids, proteins, drugs, and environmental toxins. Secondly, the failure of the liver to produce normal bile acids may lead to the accumulation of intermediary metabolites, which may contribute to the liver injury [22]. The combination of both-PiZZ genotype and defect in bile acid metabolism may be a strong driver of liver fibrosis early in childhood. More than 30 years ago, Nemeth and co-authors investigated the serum and urinary bile acid patterns of infants and PiZZ AATD children with different natural courses. The authors found that children with increased polyhydroxilation of bile acids, which make them more hydrophilic, have a benign natural course and did not develop chronic liver disease. In contrast, those infants who showed synthesis of more primitive forms of bile acids, such as bile alcohols, developed early liver fibrosis and cirrhosis. Due to the small number of patients and because of the analytical limitations, these findings were regarded more as an indication than a proof of concept [23,24]. In general, hydrophobic bile acids are cytotoxic and the biliary HCO3- umbrella hypothesis suggests that human cholangiocytes and hepatocytes create a protective apical alkaline barrier [25]. Therefore, PiZZ AATD children who develop liver disease may have an impaired biliary HCO3- umbrella leading to defective lipid metabolism, uncontrolled exposure to toxic bile acids, and cell apoptosis and senescence. Current therapies stabilizing presumed defective biliary HCO3- umbrellas in PiZZ AATD children would be of interest to explore.

To continue the search for any putative genes and related pathways, which can help to characterize the clinical outcome of the PiZZ child’s liver, we analyzed liver biopsies for the expression of fibrosis, as well as eight bile acid metabolism-related genes. 

Hepatic fibrogenesis is a complex process involving numerous extracellular matrix components such as collagen, matrix metalloproteinases, and metallopeptidase inhibitors (TIMPs) as well as regulatory factors like TGF-β or platelet derived growth factor beta (PDGF-β) [26]. When compared to NCH or NNCH, livers of NCC children showed significantly upregulated genes associated with liver fibrosis and cirrhosis. Specifically, COL4A1 and COL4A2 expression was higher in NCC than in NCH or NNCH groups. On the level of biological pathways, we found increased activity of ECM-related pathways involved in collagen biosynthesis, ECM organization, and the regulation of metallopeptidase activity. Likewise, other studies reported significantly upregulated expression of COL4A1 and COL4A2 in patients with liver cirrhosis and hepatocellular carcinoma [27]. Collagen genes in general activate intracellular signaling events to promote cell survival, proliferation, and tumorigenesis, and are involved in immune infiltration and epithelial–mesenchymal transition [28]. In parallel, genes like VIM and THBS1 were also significantly upregulated in NCC relative to NCH or NNCH. VIM is a type of intermediate filament protein that contributes to cell migration, adhesion, inflammation and apoptosis [29], and is found to be significantly upregulated in advanced liver fibrosis [30] since it is expressed in a wide range of non-epithelial cells. Like VIM, THBS1 is a marker of liver fibrosis and cirrhosis with important roles in inflammation, cellular adhesion, growth, migration, and angiogenesis [31,32]. It is suggested that THBS1 contributes to liver fibrosis both as an activator of TGFβ and as a modulator of angiogenesis [32]. TIMP1 has also been suggested as a promoter of hepatic fibrosis through the inhibition of matrix degradation [33]. We previously reported higher plasma levels of TIMP-1 in young adults with PiZ AATD relative to non-deficient, matched controls [34]. The higher expression of TIMP1 in NCC than in NCH or NNCH further confirms the importance of TIMPs/MMPs in the pathogenesis of AATD-related liver disease. Taken together, PiZZ children with NCC showed higher expression of several genes and upregulated pathways related to liver fibrosis/cirrhosis, relative to NCH or NNCH PiZZ children. However, the upregulation of these genes most likely is a result, not a cause, of liver damage.

Recently, researchers engineered PiZZ pluripotent stem cell-derived hepatocytes (iHeps) [35], which recapitulated previously reported endoplasmic reticulum stress and mitochondrial dysfunction, among other defects in transcriptomic and metabolomic pathways. Specifically, urea cycle metabolites were found significantly altered in PiZZ iHeps, which was associated with downregulation of urea cycle enzymes (ASS1, CPS1, OTC, ASL), which supports previous observations in PiZ mice regarding ureagenesis impairment [36]. Other studies also proposed that PiZZ individuals might have an impaired ability to detoxify ammonia [6]. One of the above-mentioned urea cycle enzymes encoding genes, namely ASL, which encodes the subunit of argininosuccinate lyase (EC 4.3.2.1), was included in our analyzed gene panel. We found that PiZZ children with NCC showed significantly higher expression of ASL compared to NCH or NNCH. The upregulation of ASL or other urea cycle enzymes during the end-stage liver disease might be a compensatory mechanism to secure sufficient detoxification of ammonia. Further studies are warranted to investigate in depth the role of the urea cycle in PiZZ livers.

On the other hand, specific genes and metabolic pathways were downregulated in the NCC group. When compared to NCH and NNCH, the NCC group showed significantly lower expression of AKR1D1 and numerous ADH genes in line with decreased activity of several aldehyde- and ketone-related biological pathways. Aldo-keto reductase family 1 (AKR1) is a superfamily of proteins that participates in converting aldehydes and ketones to their corresponding alcohols by utilizing NADH or NADPH, redox co-factors that play an important role in hepatic metabolism [7,37]. The hepatic AKR1D1 governs bile acids production and generates all 5β-reduced metabolites for C19-, C21- and C27-steroids, including androgens and glucocorticoids. Several studies showed that the dysregulation/deficiency of AKR1D1 might lead to chronic liver disease [38,39]. This finding is in line with earlier ideas that PiZZ liver disease might be associated with errors in bile acid metabolism, as discussed above.

It is also important to point out that several genes belonging to the cytochrome P-450 (CYP) family showed significantly lower expression in NCC relative to NCH or NNCH groups. The CYP enzymes are mainly expressed in the liver and are involved in mono-oxygenation and hydroxylation of steroids, bile acids, prostaglandins, and leukotrienes as well as xenobiotics such as drugs and alcohol. CYP1A2 is one of the major enzymes of the cytochrome P450 complex. Its content and activity have been described to be highly influenced by various hepatic diseases and several studies could demonstrate a progressive CYP1A2 decrease following experimental bile duct ligation in mouse and rat models [40,41]. In light of these studies, the reduced hepatic CYP1A2 transcript levels observed in NCC group appear comprehensible. Previous studies reported evidence for CYP2E1 as a marker of hepatocyte differentiation and a contributor to the pathogenesis of non-alcoholic steatohepatitis and hepatic cirrhosis due to its association with lipid peroxidation and the production of reactive oxygen species, as well as consecutive damage to cellular and mitochondrial membranes [42]. Likewise, we found lower levels of CYP2E1 transcript in HCC relative to groups with a favourable outcome.

The farnesoid X receptor (NR1H4), a bile acid-activated transcription factor, is essential for bile acid homeostasis [43]. When bound to bile acids, this protein binds to specific DNA motifs and regulates the expression of genes involved in bile acid synthesis and transport [44]. NR1H4 deficiency has been associated with progressive intrahepatic cholestasis and subsequent hepatic fibrosis [45]. NR1H3, also known as liver X receptor α (LXR-α), is an important regulator for the expression of multiple enzymes and transporters involved in cholesterol and fatty acid homeostasis via heterodimerization with the retinoid X receptor (RXR) [46]. Data from experimental acute liver disease models suggest that NR1H3 performs anti-inflammatory and anti-fibrotic functions [47,48]. According to our data, NR1H4 and NR1H3 levels as well as activity of the bile acid catabolism pathway are lower in NCC than in NCH and NNCH PiZZ children.

Furthermore, the NCC group showed lower expression of HMGCS2 and ACOX2 genes relative to the NCH or NNCH groups. The HMGCS2 gene encodes a mitochondrial enzyme that catalyzes the first reaction of ketogenesis, one of the major metabolic pathways. HMGCS2 is considered as the key rate-limiting enzyme of ketogenesis [49]. In times of carbohydrate deprivation (e.g., fasting), this pathway provides lipid-derived energy. ACOX2 encodes a peroxisomal enzyme, which is a member of the acyl-CoA oxidase family and involved in the degradation of long-branched fatty acids and bile acid intermediates [50]. The expression of another gene involved in cholesterol metabolism, namely EPHX2, was also lower in the NCC group. 

Taken together, the perturbations in expression of the genes related to lipid, cholesterol, and bile acid homeostasis seem to reflect the severe course of liver disease in PiZZ children. The main limitation of this study is the relatively small size of the retrospective cohort. Moreover, the small sizes of the liver biopsies did not permit investigations that are more comprehensive. These latter limitations restricted scientists to perform similar studies previously. As far as we know, this is the first retrospective study investigating liver Z-AAT polymers and fibrosis-related gene expression in children with different clinical presentations of AATD related liver disease. Our data provide clear evidence that Z-AAT polymers per se cannot explain clinical outcomes of the liver in PiZZ AATD children. On the other hand, changes in the expression of specific genes and pathways involved in lipid, steroid, and bile acid homeostasis and metabolism, appear to be involved in the degree of liver injury and warrant further investigations.

## 4. Materials and Methods

### 4.1. Patients

From 14 PiZZ AATD children we obtained clinical diagnoses, including follow-up data, and liver tissues samples collected as a part of the routine clinical work-up during 1979–2010. Formalin fixed and paraffin embedded (FFPE) liver biopsies were stored according to the bio-bank regulations of Stockholm’s Karolinska University Hospital Biobank Council. The specimens were fully de-identified, with only the specimen number and diagnosis known. Only one of the investigators (A.N.) knows the identity of the patients. This study’s ID is K2018-1428, approved by the Karolinska University Hospital Ethics Committee 2018/754-31/2. According to the standardized hospital protocols, AAT concentration was determined by serum protein capillary electrophoresis, phenotyping was determined by isoelectric focusing, and biochemical liver function tests were performed. For the patients older than 1 year, serum fasting bile acids were analysed to facilitate the diagnosis of cholestasis. For all children, annual health check-ups were performed up to their adulthood. Liver biopsies were taken at varying ages, depending on the clinical course.

### 4.2. Clinical Diagnosis of PiZZ AATD Children

Neonatal cholestasis was defined as evidence of clinical jaundice, acholic stools, steatorrhea, or pruritus, and based on biochemical signs, including elevated total bilirubin levels and the conjugated being not less than 20% of the total bilirubin in infants below 4 months of age. At 2–4 months of age, a routine clinical work-up was performed: all known microbial causes of neonatal cholestasis were looked for (HAV, HBV, HCV, TORCH: toxoplasma, other viruses, e.g., EBV, rubella, and CMV). In addition, during these years the infants were screened for a growing number of inborn errors of metabolism. Unfavourable liver outcome was suspected in children who either had threatening clinical signs, such as failure to thrive, hepatosplenomegaly, or low serum concentrations of markers of hepatic synthetic capacity. Portal hypertension, indicated by thrombocytopenia and a progressive splenomegaly, was confirmed by abdominal ultrasonography. In these cases, liver cirrhosis was ruled out or confirmed based on biopsy findings. The 14 PiZZ children included were divided into three groups, according to the natural course of the liver disease. Main clinical and diagnostic characteristics are presented in Table 1. For a detailed description of patient groups, see supplementary materials and methods, and Supplementary Table S1.

### 4.3. Liver Biopsies of PiZZ AATD Children

Percutaneous liver biopsies performed according to Menghini [11] were immediately fixed in formalin and embedded in paraffin. Two senior pathologists investigated liver tissues blindly for fibrosis, steatosis, necrosis, and PAS-Diastase (PAS-D) positive globules. Fibrosis was defined by the staining with Sirius or Reticulin and, in some early cases, extra- and intrahepatic cholestasis was investigated in the Fe-stained samples. Proliferating bile ducts, visualized by anti-cytokeratin CK19 or 7 staining, were regarded as additional histological signs of cholestasis. Inflammation was noticed, both intralobular and in the portal zones. Fibrosis was scored according to Batts and Ludwig [51]. PAS-D accumulation was scored as PAS-D- (no cells with inclusions), PAS-D+ (<5 cells/biopsy), PAS-D++ (5–20 cells/biopsy), or PAS-D+++ (>20 cells/biopsy). Representative images of PAS-D staining are shown in Figure 1. As expected, the PAS-D globules were found almost exclusively in the periportal areas, but their number could vary greatly even in adjacent hepatocytes. This last makes impossible a realistic quantification of PAS-D.

### 4.4. Immunohistochemistry

FFPE liver tissue sections were deparaffinized in two steps with xylene and xylene and isopropanol (1:1 ratio), respectively, followed by hydration with ethanol gradient in a descending order (100%, 90%, and 70%, respectively). Antigen retrieval was achieved by heat-induced epitope retrieval (HIER) method using 10 mM citric acid buffer (pH 6.0). Endogenous peroxidases were blocked with 3% H_2_O_2_ for 10 min at RT and unspecific antigens were blocked with 10% fetal calf serum in 0.3% Tween-20 in PBS for 1 h. Post blocking, tissue sections were incubated with mouse monoclonal anti-Z AAT polymers D11 at 1:800 dilution, at 4 °C, overnight. Detection of bound antibodies was carried out using One step HRP-polymer (Cat#GTX83398, Gene Tex, Wien, Austria) followed by 3,3′-Diaminobenzidine (DAB) staining. Hematoxylin was used as a counterstain. Sections were dehydrated in ascending alcohol gradient (70%, 90%, and 100%, respectively) and finally submerged in xylene. Thereafter, tissue sections were covered with coverslips using Eukitt mounting medium (Cat# 25608-33-7, Sigma Aldrich, St. Louis, MO, USA). Images were taken at 100x magnification using a Leica DM750 microscope equipped with a Leica ICC50HD camera (Leica, Wetzlar, Germany). Three independent examiners evaluated all samples.

### 4.5. Gene Expression, Biological Pathways, and Statistics

Due to the small sample size, each liver biopsy was analysed in duplicates using FFPE samples mounted on glass slides. The isolation of RNA, quality control, and NanoString analyses were performed by Canopy Biosciences, St. Louis, MO, USA (https://canopybiosciences.com/sample-guidelines-nanostring-services, accessed on 16 October 2020. Differential gene expression between groups was analysed using the nSolver 4.0 software (NanoString Technologies, Seattle, WA, USA). The ascertained log2 mRNA counts were analysed using R software version 3.4.4 (R Foundation for Statistical Computing, Vienna, Austria) and the nCounter Advanced Analysis module version 1.1.5 (NanoString Technologies). Normality was tested using the Shapiro-Wilks and Kolmogorov-Smirnov tests. U-tests were used for pairwise comparisons between groups, and statistical analysis of gene expression included correction for multiple testing using the Benjamini-Hochberg method. Adjusted *p*-values <0.05 were statistically significant. Biological pathway analysis was performed using the Rosalind platform accessible online via https://app.rosalind.bio/ and the .rcc files supplied by NanoString as input data. The data on the regulation of biological processes were calculated using a standardized hypergeometric distribution algorithm and the Ontology collection. Given the small sample size due to the rareness of available tissue samples, a hierarchical cluster analysis was performed to confirm consistency within groups and difference between groups using the ward method for groups.

## Figures and Tables

**Table 1:** Clinical and diagnostic characteristics of PiZZ children.

Patient Group	Gender	Age at Biopsy (Years)	Clinical Status (Last Contact)	Liver Function (Last Contact)	Other Organs	Hepatocytes	Fibrosis	Portal Tract		Inflammation	Fibrosis (Total)	F/4 (After Tx)
								Fibrosis	Bile Duct Proliferation			
NCH	F	10	0	0	0	PAS-D+		++	++		+	
	F	1	0	0	0	PAS-D++		++	++		(+)	
	M	4	0	0	0	PAS-D+++		++	++		++	
	F	5	0	0	0	PAS-D+		+++	+++	+	++	
	M	8	0	0	0	PAS-D+++		+	+		+	
NCC	M	3	MOF	failure	MOF	PAS-D+ steatosis	+	++++	+		++++	Tx
	F	10	MOF	failure	MOF	PAS-D +	+++	++++	++		++++	Tx
	F	5	MOF	failure	MOF	PAS-D +		++++	+++	+	++++	Tx
	F	15	MOF	failure	MOF	PAS-D -	++	++++	+++		++++	Tx
NNCH	M	2	0	0	0	PAS-D++		++	+		++	
	F	4	0	0	0	PAS-D++		+	+		+	
	F	12	0	0	0	PAS-D-			+			
		19				PAS-D+			+			
	M	6	0	0	0	Vacuoli					+	
	M	4	0	0	0	PAS-D-		+				

F, female; M, male; MOF, multiple organ failure; PAS-D, Periodic acid Schiff plus diastase; Tx, transplantation; Vacuoli, vacuoles in hepatocytes indicates a marked fat accumulation. 0: indicates the absence of clinical symptoms and/or biochemical evidence of hepatic or extrahepatic organ damage. PAS-D accumulation was scored as PAS-D- (no cells with inclusions), PAS-D+ (<5 cells/biopsy), PAS-D++ (5–20 cells/biopsy), or PAS-D+++ (>20 cells/biopsy). Fibrosis score: + mild; ++ moderate, +++ severe, ++++ very severe.

## Data Availability

The data presented in this study are available on request from the corresponding author.

## Supplementary Materials

The following supporting information can be downloaded at https://www.mdpi.com/article/10.3390/ijms24032485/s1.

## References

[1] Janciauskiene S.M., Bals R., Koczulla R., Vogelmeier C., Kohnlein T., Welte T. (2011). The discovery of alpha1-antitrypsin and its role in health and disease. Respir. Med..

[2] Blanco I., de Serres F.J., Fernandez-Bustillo E., Lara B., Miravitlles M. (2006). Estimated numbers and prevalence of PI*S and PI*Z alleles of alpha1-antitrypsin deficiency in European countries. Eur. Respir. J..

[3] Stoller J.K., Smith P., Yang P., Spray J. (1994). Physical and social impact of alpha 1-antitrypsin deficiency: Results of a survey. Clevel. Clin. J. Med..

[4] Sveger T. (1976). Liver disease in alpha1-antitrypsin deficiency detected by screening of 200,000 infants. N. Engl. J. Med..

[5] Sveger T. (1978). Alpha 1-antitrypsin deficiency in early childhood. Pediatrics.

[6] Sveger T. (1988). The natural history of liver disease in alpha 1-antitrypsin deficient children. Acta Paediatr. Scand..

[7] Katzer D., Ganschow R., Strnad P., Hamesch K. (2021). Pi*ZZ-related liver disease in children and adults—Narrative review of the typical presentation and management of alpha-1 antitrypsin deficiency. Dig. Med. Res..

[8] Suri A., Patel D., Teckman J.H. (2022). Alpha-1 Antitrypsin Deficiency Liver Disease. Clin. Liver Dis..

[9] Wu Y., Whitman I., Molmenti E., Moore K., Hippenmeyer P., Perlmutter D.H. (1994). A lag in intracellular degradation of mutant alpha 1-antitrypsin correlates with the liver disease phenotype in homozygous PiZZ alpha 1-antitrypsin deficiency. Proc. Natl. Acad. Sci. USA.

[10] Sveger T. (1984). Prospective study of children with alpha 1-antitrypsin deficiency: Eight-year-old follow-up. J. Pediatr..

[11] Nebbia G., Hadchouel M., Odievre M., Alagille D. (1983). Early assessment of evolution of liver disease associated with alpha 1-antitrypsin deficiency in childhood. J. Pediatr..

[12] Francavilla R., Castellaneta S.P., Hadzic N., Chambers S.M., Portmann B., Tung J., Cheeseman P., Rela M., Heaton N.D., Mieli-Vergani G. (2000). Prognosis of alpha-1-antitrypsin deficiency-related liver disease in the era of paediatric liver transplantation. J. Hepatol..

[13] Townsend S.A., Edgar R.G., Ellis P.R., Kantas D., Newsome P.N., Turner A.M. (2018). Systematic review: The natural history of alpha-1 antitrypsin deficiency, and associated liver disease. Aliment. Pharmacol. Ther..

[14] Volpert D., Molleston J.P., Perlmutter D.H. (2000). Alpha1-antitrypsin deficiency-associated liver disease progresses slowly in some children. J. Pediatr. Gastroenterol. Nutr..

[15] Khodayari N., Wang R.L., Oshins R., Lu Y., Millett M., Aranyos A.M., Mostofizadeh S., Scindia Y., Flagg T.O., Brantly M. (2021). The Mechanism of Mitochondrial Injury in Alpha-1 Antitrypsin Deficiency Mediated Liver Disease. Int. J. Mol. Sci..

[16] Bouchecareilh M. (2020). Alpha-1 Antitrypsin Deficiency-Mediated Liver Toxicity: Why Do Some Patients Do Poorly? What Do We Know So Far?. Chronic Obstr. Pulm Dis..

[17] Marek G., Collinsworth A., Liu C., Brantly M., Clark V. (2021). Quantitative measurement of the histological features of alpha-1 antitrypsin deficiency-associated liver disease in biopsy specimens. PLoS ONE.

[18] Noordeen F. (2015). Hepatitis B virus infection: An insight into infection outcomes and recent treatment options. Virusdisease.

[19] Tseng T.C., Huang L.R. (2017). Immunopathogenesis of Hepatitis B Virus. J. Infect. Dis..

[20] Lindblad D., Blomenkamp K., Teckman J. (2007). Alpha-1-antitrypsin mutant Z protein content in individual hepatocytes correlates with cell death in a mouse model. Hepatology.

[21] Schaefer B., Haschka D., Finkenstedt A., Petersen B.S., Theurl I., Henninger B., Janecke A.R., Wang C.Y., Lin H.Y., Veits L. (2015). Impaired hepcidin expression in alpha-1-antitrypsin deficiency associated with iron overload and progressive liver disease. Hum. Mol. Genet..

[22] Chiang J.Y.L., Ferrell J.M. (2018). Bile Acid Metabolism in Liver Pathobiology. Gene Expr..

[23] Nemeth A., Strandvik B. (1982). Natural history of children with alpha 1-antitrypsin deficiency and neonatal cholestasis. Acta Paediatr. Scand..

[24] Nemeth A., Strandvik B. (1984). Urinary excretion of tetrahydroxylated bile acids in children with alpha 1-antitrypsin deficiency and neonatal cholestasis. Scand. J. Clin. Lab. Investig..

[25] Beuers U., Hohenester S., de Buy Wenniger L.J., Kremer A.E., Jansen P.L., Elferink R.P. (2010). The biliary HCO(3)(-) umbrella: A unifying hypothesis on pathogenetic and therapeutic aspects of fibrosing cholangiopathies. Hepatology.

[26] Lefeuvre C., Roux M., Blanchard S., Le Guillou-Guillemette H., Boursier J., Lunel-Fabiani F., Jeannin P., Pivert A., Ducancelle A. (2022). Analysis of hepatic fibrosis markers in the serum of chronic hepatitis B patients according to basal core promoter/precore mutants. Sci. Rep..

[27] Liu Y., Zhang J., Chen Y., Sohel H., Ke X., Chen J., Li Y.X. (2020). The correlation and role analysis of COL4A1 and COL4A2 in hepatocarcinogenesis. Aging.

[28] Shin H.J., Gil M., Lee I.S. (2022). Association of Elevated Expression Levels of COL4A1 in Stromal Cells with an Immunosuppressive Tumor Microenvironment in Low-Grade Glioma, Pancreatic Adenocarcinoma, Skin Cutaneous Melanoma, and Stomach Adenocarcinoma. J. Pers. Med..

[29] Paulin D., Lilienbaum A., Kardjian S., Agbulut O., Li Z. (2022). Vimentin: Regulation and pathogenesis. Biochimie.

[30] Wang W.M., Zhang W.S., Yang Z.G. (2022). Vimentin (VIM) predicts advanced liver fibrosis in chronic hepatitis B patients: A random forest-derived analysis. Eur. Rev. Med. Pharmacol. Sci..

[31] Min-DeBartolo J., Schlerman F., Akare S., Wang J., McMahon J., Zhan Y., Syed J., He W., Zhang B., Martinez R.V. (2019). Thrombospondin-I is a critical modulator in non-alcoholic steatohepatitis (NASH). PLoS ONE.

[32] Li Y., Turpin C.P., Wang S. (2017). Role of thrombospondin 1 in liver diseases. Hepatol. Res..

[33] Iredale J.P. (1997). Tissue inhibitors of metalloproteinases in liver fibrosis. Int. J. Biochem. Cell Biol..

[34] Janciauskiene S., Subramaniyam D., Piitulainen E., Kohnlein T., Sveger T. (2010). Plasma levels of TIMP-1 are higher in 34-year-old individuals with severe alpha1-antitrypsin deficiency. Thorax.

[35] Kaserman J.E., Werder R.B., Wang F., Matte T., Higgins M.I., Dodge M., Lindstrom-Vautrin J., Hinds A., Bullitt E., Caballero I.S. (2022). Modeling of Alpha-1 Antitrypsin Deficiency with Syngeneic Human iPSC-Hepatocytes Reveals Metabolic Dysregulation and Cellular Heterogeneity in PiMZ and PiZZ Hepatocytes. bioRxiv.

[36] Piccolo P., Annunziata P., Soria L.R., Attanasio S., Barbato A., Castello R., Carissimo A., Quagliata L., Terracciano L.M., Brunetti-Pierri N. (2017). Down-regulation of hepatocyte nuclear factor-4alpha and defective zonation in livers expressing mutant Z alpha1-antitrypsin. Hepatology.

[37] Goodman R.P., Markhard A.L., Shah H., Sharma R., Skinner O.S., Clish C.B., Deik A., Patgiri A., Hsu Y.H., Masia R. (2020). Hepatic NADH reductive stress underlies common variation in metabolic traits. Nature.

[38] Nikolaou N., Gathercole L.L., Marchand L., Althari S., Dempster N.J., Green C.J., van de Bunt M., McNeil C., Arvaniti A., Hughes B.A. (2019). AKR1D1 is a novel regulator of metabolic phenotype in human hepatocytes and is dysregulated in non-alcoholic fatty liver disease. Metabolism.

[39] Nikolaou N., Gathercole L.L., Kirkwood L., Dunford J.E., Hughes B.A., Gilligan L.C., Oppermann U., Penning T.M., Arlt W., Hodson L. (2019). AKR1D1 regulates glucocorticoid availability and glucocorticoid receptor activation in human hepatoma cells. J. Steroid Biochem. Mol. Biol..

[40] Abshagen K., Konig M., Hoppe A., Muller I., Ebert M., Weng H., Holzhutter H.G., Zanger U.M., Bode J., Vollmar B. (2015). Pathobiochemical signatures of cholestatic liver disease in bile duct ligated mice. BMC Syst. Biol..

[41] Fukushima S., Okuno H., Shibatani N., Nakahashi Y., Seki T., Okazaki K. (2008). Effect of biliary obstruction and internal biliary drainage on hepatic cytochrome P450 isozymes in rats. World J. Gastroenterol..

[42] Villeneuve J.P., Pichette V. (2004). Cytochrome P450 and liver diseases. Curr. Drug Metab..

[43] Keitel V., Droge C., Haussinger D. (2019). Targeting FXR in Cholestasis. Handb Exp. Pharmacol..

[44] Ramos Pittol J.M., Milona A., Morris I., Willemsen E.C.L., van der Veen S.W., Kalkhoven E., van Mil S.W.C. (2020). FXR Isoforms Control Different Metabolic Functions in Liver Cells via Binding to Specific DNA Motifs. Gastroenterology.

[45] Czubkowski P., Thompson R.J., Jankowska I., Knisely A.S., Finegold M., Parsons P., Cielecka-Kuszyk J., Strautnieks S., Pawlowska J., Bull L.N. (2021). Progressive familial intrahepatic cholestasis—Farnesoid X receptor deficiency due to NR1H4 mutation: A case report. World J. Clin. Cases.

[46] Hong C., Tontonoz P. (2014). Liver X receptors in lipid metabolism: Opportunities for drug discovery. Nat. Rev. Drug Discov.

[47] Beaven S.W., Wroblewski K., Wang J., Hong C., Bensinger S., Tsukamoto H., Tontonoz P. (2011). Liver X receptor signaling is a determinant of stellate cell activation and susceptibility to fibrotic liver disease. Gastroenterology.

[48] Hamilton J.P., Koganti L., Muchenditsi A., Pendyala V.S., Huso D., Hankin J., Murphy R.C., Huster D., Merle U., Mangels C. (2016). Activation of liver X receptor/retinoid X receptor pathway ameliorates liver disease in Atp7B(-/-) (Wilson disease) mice. Hepatology.

[49] Asif S., Kim R.Y., Fatica T., Sim J., Zhao X., Oh Y., Denoncourt A., Cheung A.C., Downey M., Mulvihill E.E. (2022). Hmgcs2-mediated ketogenesis modulates high-fat diet-induced hepatosteatosis. Mol. Metab..

[50] Alonso-Pena M., Espinosa-Escudero R., Herraez E., Briz O., Cagigal M.L., Gonzalez-Santiago J.M., Ortega-Alonso A., Fernandez-Rodriguez C., Bujanda L., Calvo Sanchez M. (2022). Beneficial effect of ursodeoxycholic acid in patients with acyl-CoA oxidase 2 (ACOX2) deficiency-associated hypertransaminasemia. Hepatology.

[51] Batts K.P., Ludwig J. (1995). Chronic hepatitis. An update on terminology and reporting. Am. J. Surg. Pathol..

