# Supplementary material for: Fibrosis-Related Gene Profiling in Liver Biopsies of PiZZ α1-Antitrypsin Children with Different Clinical Courses
Source: Int J Mol Sci. 2023 Jan 27;24(3):2485. doi: 10.3390/ijms24032485 (PMC9916468; doi:10.3390/ijms24032485)
Supplement: Supplementary file 1 [file ijms-24-02485-s001.zip › Supplementary materials.pdf]

Supplementary Materials and Methods

Patient characterization

The included 14 PiZZ children were divided into 3 groups, according to the natural course of the liver disease. To minimize confounding factors, patients with unspecific risk factors, as malnutrition, severe intra- or extrauterine bacterial infections were excluded.

The first group, named as NCH, included 5 children who had neonatal cholestasis but did not develop chronic liver disease. These patients were referred to the tertiary liver center during their infancy due to suspicion of liver disease. Liver biopsy was performed early during the follow-up but at varying ages, depending on the clinical course. After this initial work-up the children were followed-up periodically until they reached adolescent age. During childhood and adolescence these children developed normally and had no signs of organ dysfunction and/or hepatosplenomegaly. In most of these children serum transaminases remained slightly elevated for some years, with AST and ALT always below three times of the upper normal level. The function of all organs was normal.

The second group, named as NCC, included 4 children who at varying ages developed neonatal cholestasis and chronic progressive liver disease leading to liver transplantation. We were able to obtain at least one good-quality biopsy before liver transplantation, however their postoperative follow-up is not a part of this report.

The third group, named as NNCH, included 5 children (two biopsies were included from one female at age of 12 and 19 years) who did not develop neonatal cholestasis or signs of liver damage and remained healthy. These children have been found either as healthy siblings to sick children, or through national screening, running at the time of their birth, or else have been found accidentally. Once children were diagnosed as PiZZ, they participated in the follow-up program, and since the clinical impact of the AATD was initially unknown, in few cases liver biopsies were performed. For children belonging to NCH and NNCH groups the shortest follow-up period after liver biopsy was 5 years. At the check-ups, complete physical assessment including investigation of pubertal development took place and hepatosplenomegaly was excluded.

NanoString gene panel

Samples were analyzed using the human pan fibrosis plus panel (NanoString Technologies, Seattle, WA) on 760 different fibrosis-specific genes complemented by 8 self-selected genes of interest: G-protein-coupled BA receptor (TGR5), aldo-keto reductase family 1 member D1 (AKR1D1), hydroxy-delta-5-steroid dehydrogenase 3 beta 1 and 3 beta 7 (HSD3B1 and HSD3B7), carbamoyl-phosphate synthetase 1 (CPS1), ornithine transcarbamylase (OTC), argininosuccinate synthetase (ASS1), and argininosuccinate lyase (ASL). Counts were normalized using the nSolver analysis software version 4.0 (NanoString Technologies, Seattle, WA). Nucleolar protein 7 (NOL7), ribosomal protein lateral stalk subunit P0 (RPLP0), glucuronidase beta (GUSB), armadillo like helical domain containing 3 (ARMH3), peptidylprolyl isomerase A (PPIA), myotubularin related protein 14 (MTMR14), phosphoglycerate kinase 1 (PGK1), nucleotide binding protein 1 (NUBP1), acyl-CoA dehydrogenase family member 9 (ACAD9), and CCR4-NOT transcription complex subunit 10 (CNOT10) were used as reference genes for normalization

Supplementary Table S1: Laboratory parameters of ZZ AATD cases

Patient Group	Gender	Age (years)	Date of analysis	ASAT (µkata /L)	ALAT (µkata /L)	ASAT/ALAT	ALP µkata/L	Bilirubin/s (µM/L)	γ–GT µkatal/L	Platelets
NCH	F	10	02.03.22	0.91	1.31	0.69	2.6	8	0.28	>150.000
	F	1	05.03.22	2.93	3.01	0.97	2.7	3	0.37	>150.000
	M	4	06.11.24	1.09	1.76	0.62	4.5	5	0.25	>150.000
	F	5	09.03.24	1.67	1.75	0.95	4.2	9/2	0.57	>150.000
	M	8	07.05.25	0.69	0.49	1.41	4.2	10/3	0.41	>150.000
NCC	M	3	89-12-15	4.04	3.56	1.13	17.6	9	7.74	>150,000
	F	10	01.08.21	2.38	1.72	1.38	12.1	27/12	2.57	44.000
	F	5	93-03-08	3.1	3.05	1.02	11.5	13	8.73	>150.000
	F	15	87-05-07	1.5	1.30	1.15	-	73	1.30	65.000
NNCH	M	2	09.11.11	1.22	0.95	1.28	3.7	3	0.27	>150.000
	F	4	11.05.09	1.13	0.86	1.31	3.8	6/1	0.21	>150.000
	F	12	81-08-24	0.46	0.61	0.75	-	2	1.97	120.000
		19	88-09-22	-	-	-	-	2		>150.000
	M	6	80-02-07	0.98	1.52	0.64	7.37	3	0.17	>150.000
	M	4	79-08-29	1.11	0.94	1.18	8.13	<1	0.30	>150.000
	References:			<0.7	<0.7		<5.0	<10.0	<0.50	>150.000

Supplementary Table S2: Biological functions.

		NCC vs NCH			NCH vs NNCH			NCC vs NNCH		
**Pathway**	**# of genes**	**p-value**	**# up**	**# down**	**p_value**	**# up**	**# down**	**p_value**	**# up**	**# down**
Acyl-CoA metabolic process	105	0.300	0	2	0.170	2	0	0.010	5	0
Alcoholic catabolic process	54	0.132	0	2	0.633	1	0	0.005	0	2
Aldehyde catabolic process	11	0.020	0	1	-	-	-	0.020	0	1
Bile acid catabolic process	3	0.020	0	1	-	-	-	0.020	0	1
Cholesterol metabolic process	150	0.034	1	7	0.307	7	0	0.525	0	1
Collagen biosynthetic process	53	0.056	4	0	0.905	1	0	0.029	2	0
ECM organization	368	0.050	11	3	0.744	11	0	0.003	6	0
Epoxygenase P450 pathway	20	<0.001	0	6	0.109	3	0	0.009	0	2
Ethanol oxidation	12	<0.001	0	4	0.487	1	0	0.002	0	2
Exogenous drug catabolic process	24	<0.001	0	5	0.077	3	0	0.007	0	2
Fatty acid metabolic process	383	0.047	1	14	0.395	9	1	0.585	0	3
Lipid homeostasis	151	0.005	0	9	0.750	4	0	-	0	0
Lipid hydroxylation	7	0.033	0	2	0.963	2	0	0.059	0	1
Lipid metabolic process	1407	0.596	3	25	0.009	31	4	0.885	0	6
Long-chain fatty acid biosynthetic process	30	0.006	0	4	0.109	3	0	0.009	0	2
Monoterpenoid metabolic process	6	0.005	0	3	0.487	1	0	0.002	0	2
Negative regulation of metallopeptidase activity	14	0.012	2	0	0.283	1	0	0.001	2	0
Omega-hydroxylase P450 pathway	9	0.011	0	3	0.566	1	0	0.096	0	1
Positive regulation of collagen biosynthetic process	26	0.011	3	0	-	0	0	0.096	1	0

Positive regulation of collagen metabolic process	27	0.011	3	0	-	0	0	0.096	1	0
Regulation of lipid transport	127	0.608	1	2	0.946	2	0	0.020	0	1
Regulation of mitochondrion organization	182	0.006	2	5	0.615	4	0	-	0	0
Retinoic acid metabolic process	32	0.001	0	4	0.170	2	0	0.096	0	1
Retinol metabolic process	41	<0.001	0	5	0.689	1	0	<0.001	0	3
Steroid catabolic process	27	0.132	0	2	0.633	1	0	0.005	0	2
Steroid metabolic process	331	0.002	2	13	0.164	11	2	0.655	0	3
Xenobiotic metabolic process	125	0.001	0	6	0.320	3	0	0.026	0	2

ECM, extracellular matrix.
